# Angiomyolipoma of the Right Adrenal Gland

**DOI:** 10.5402/2011/102743

**Published:** 2011-04-18

**Authors:** Oktay Yener, Alp Özçelik

**Affiliations:** Department of Surgery, Göztepe Training and Research Hospital, İstanbul 81060, Turkey

## Abstract

Adrenal angiomyolipoma is rare. Only four cases have been reported so far. These are commonly found in Kidney but extrarenal sites are also mentioned. Angiomyolipoma arising in adrenal is very rare entity, usually asymptomatic, diagnosed incidentally on radiological investigation of abdomen for other conditions. We report our experience with a 45-year-old woman who presented with epigastric discomfort. A computerised tomography (CT) scan showed an adrenal mass. Laparoscopic adrenalectomy was performed and the histopathological features confirmed the diagnosis of adrenal angiomyolipoma. The patient recovered without any complications following surgery.

Adrenal angiomyolipoma is rare. Only four cases have been reported so far. We report our experience with a 45-year-old female who presented with right subcostal pain. A computerised tomography (CT) scan showed a right adrenal mass with features of angiomyolipoma. Laparoscopic adrenalectomy was performed, and the histopathological features confirmed the diagnosis of adrenal angiomyolipoma. The patient recovered without any complications following surgery.

Angiomyolipoma is apparently a part of a family of neoplasms that derive from perivascular epithelioid cells. It is a rare mesenchymal tumor, usually found in the kidney. Extrarenal angiomyolipoma is uncommon, and the most common extrarenal site is the liver [1].

A 45-year-old female presented with epigastric discomfort off and on. Upper G.I Endoscopy was normal. Sonography for hepatobiliary system was normal but revealed a well-defined 5 × 6 cm mass in the retroperitoneum (incidentaloma) (Figure 1). CT abdomen further defined the mass as of right adrenal origin and a possibility of adrenocortical tumour.

Laboratory investigations, that is, Serum catecholamine, cortisol, and urinary, VMA were within normal limits. Exploratory laparoscopy revealed 5 × 5 × 4 cm mass, firm in consistency, quite separate from right kidney with no definable right adrenal gland. Right laparoscopic adrenalectomy was performed.

On cut section mass was grey-white and non-homogeneous in texture. Histopathological examination revealed mature fat cells, smooth muscle fibres, and thin-walled blood vessels with peripherally compressed adrenal cortical tissue suggestive of angiomyolipoma of adrenal (Figure 2). The patient made uneventful recovery and was normal at 3-month follow-up. 

Because up to 52% of patients with angiomyolipomas larger than 4 cm are symptomatic and have an increased risk of bleeding, surgery or selective arterial embolization has been suggested in large angiomyolipomas [2].

 In the last years, laparoscopic adrenalectomy has been recommended because it is less invasive, with lower mortality compared with open surgery. Management should be the same as that for any adrenal mass. Assessment of functional status of the tumor should be done. Surgery is indicated if the patient is symptomatic or the tumor is more than 5 cm since the risk of malignancy increases with size. Also, the risk of spontaneous rupture increases with size, owing to the presence of abundant and abnormal elastin-poor vascularity in the tumor. Laparoscopic adrenalectomy is an option and had been successfully done for a 5 cm adrenal angiomyolipoma [3, 4].

## Figures and Tables

**Figure 1:**
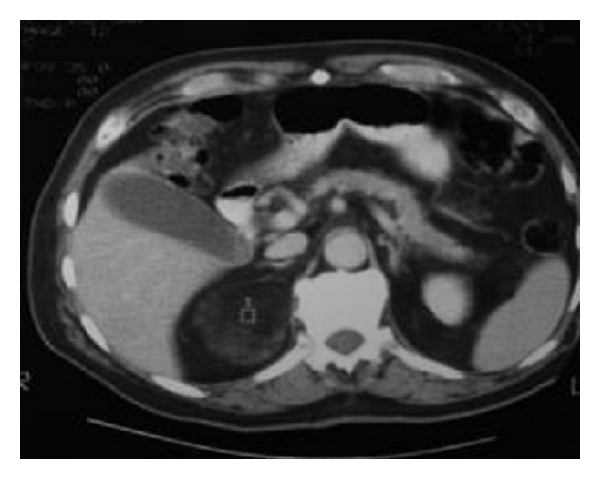
CT of the abdomen with contrast demonstrating large low-attenuation mass right adrenal gland.

**Figure 2:**
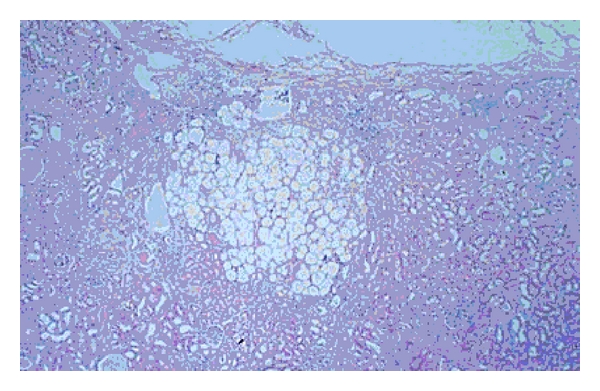
Angiomyolipoma.
